# The Systematic Bias of Entropy Calculation in the Multi-Scale Entropy Algorithm

**DOI:** 10.3390/e23060659

**Published:** 2021-05-24

**Authors:** Jue Lu, Ze Wang

**Affiliations:** 1 School of Mathematics, Physics and Information Science, Shaoxing University, Shaoxing 312000, China; admiral_lu@hotmail.com; 2 Department of Diagnostic Radiology and Nuclear Medicine, University of Maryland School of Medicine, Baltimore, MD 21201, USA

**Keywords:** entropy, multi-scale sample entropy, systematic bias

## Abstract

Entropy indicates irregularity or randomness of a dynamic system. Over the decades, entropy calculated at different scales of the system through subsampling or coarse graining has been used as a surrogate measure of system complexity. One popular multi-scale entropy analysis is the multi-scale sample entropy (MSE), which calculates entropy through the sample entropy (SampEn) formula at each time scale. SampEn is defined by the “logarithmic likelihood” that a small section (within a window of a length m) of the data “matches” with other sections will still “match” the others if the section window length increases by one. “Match” is defined by a threshold of r times standard deviation of the entire time series. A problem of current MSE algorithm is that SampEn calculations at different scales are based on the same matching threshold defined by the original time series but data standard deviation actually changes with the subsampling scales. Using a fixed threshold will automatically introduce systematic bias to the calculation results. The purpose of this paper is to mathematically present this systematic bias and to provide methods for correcting it. Our work will help the large MSE user community avoiding introducing the bias to their multi-scale SampEn calculation results.

## 1. Introduction

Complexity is an important property of a complex system such as the living organisms, Internet, traffic system etc. Measuring system complexity has long been of great interest in many research fields. Since complexity is still elusive to define, a few approximate metrics have been used to quantify complexity. One widely used measure is entropy which quantifies the irregularity or randomness. Complexity and entropy, however, diverge when complexity reaches the peak. Before the peak, complexity increases with complexity, but complexity decreases with entropy after the peak. To provide approximate solution to this dilemma, people have proposed many empirical measures. A popular one is the multi-scale entropy (MSE) proposed by Costa et al. [1]. MSE is based on Sample entropy (SampEn) [2,3], which is an extension of the well-known Approximate entropy (ApEn) [3,4] after removing the self-matching induced bias. SampEn has gained popularity in many applications such as neurophysiological data analysis [5] and functional MRI data analysis [6,7] because of the relative insensitivity to data length [2,8]. Because complex signal often presents self similarity when the signal is observed at different time scale, Costa et al first applied SampEn to the same signal but at different time scales after coarse graining. When applied to Gaussian noise and 1/f noise, it was observed that SampEn of Gaussian noise decreases with the signal subsampling scale while it stays at the same level for most of scales of a 1/f process. Since a 1/f process is known to have higher complexity (defined by the higher self similarity) than Gaussian noise, the diverging MSE of a 1/f noise and the Gaussian noise appears to support that MSE may provide an approximate approach to measure system complexity. Since its introduction, MSE has been widely used in many different applications as reflected by the thousands of paper citations [1,9]. While MSE and its variants have been shown to be effective for differentiating different system states through simulation or real data, it introduces bias by using the same threshold for identifying the repeated transit status at all time scales. Nikulin and Brismar [10] first observed that MSE not purely measures entropy but both entropy and variation at different scales. We here claimed that the changing variation captured by MSE is mainly caused by an incomplete scaling during the coarse-graining process and the subsequent variance change induced entropy change should be considered as a systematic bias to be removed.

The rest of this report is organized as follows. Section 2 is the background. To better understand the series of entropy formation, we introduced Shannon entropy, ApEn, SampEn, and MSE. Section 3 describes the bias caused by the coarse-graining process and the one threshold-for-all-scales MSE algorithm. Both a mathematical solution and a practical solution were provided to correct the bias. Section 5 concludes the paper.

## 2. Entropy and MSE

This section provides a brief history about the evolution of entropy and approximate entropy measures.

Hartley and Nyquist first used logarithm to quantify information [11,12]. Shannon then proposed the concept of Shannon entropy as a measure of information through the sum of the logarithmically weighted probability [13]. Denoting a discrete random variable by *X* and its probability by p(x), Shannon entropy of *X* is formulated as:

$$H\left(X\right)=-\sum_{x\in X}p\left(x\right)\log p(x)=E\left[\log\left(\frac{1}{p(x)}\right)\right];$$

In an analogous manner Shannon defined the entropy of a continuous distribution with the density distribution function(pdf) p(x) by:

$$H\left(X\right)=-\int_{x\in X}p(x)\log p(x)\;dx=E\left[-\log p(x)\right],$$

where *E* represent the expectation operator. Without loss of generality, in this paper we use natural logarithms to calculate entropy. When the entropy calculated via a logarithm to base *b*, it could be calculated by $H_{b}\left(X\right)=\frac{1}{\log b}H\left(X\right)$.

Shannon entropy was then extended into the Kolmogorov–Sinai(K-S) entropy [14] for characterizing a dynamic system. Assume that the F-dimension phase space is partitioned into a collection of cells of size $r^{F}$ and the state of the system is measured at constant time intervals δ. Let $p(c_{1},\ldots c_{n})$ be the joint probability that the state of system x(t=δ) is in cell $c_{1}$, x(t=2δ) is in cell $c_{2}$, … , and x(t=nδ) is in cell $c_{n}$. The K-S entropy is defined as

$$K-S\;\mathrm{entropy}=-\lim_{\delta \to 0}\lim_{r\to 0}\lim_{n\to \infty }\frac{1}{\delta n}\sum_{c_{1},\ldots ,c_{n}}p\left(c_{1},\ldots ,c_{n}\right)\log p(c_{1},\ldots ,c_{n}).$$

K-S entropy depends several parameters and is not easy to estimate. To solve this problem, Grassberger and Procaccia [15] proposed $K_{2}$ entropy as a lower bound of K-S entropy. Given a time series $U=\{u_{1},u_{2},\ldots ,u_{N}\}$ with length *N*, define a sequence of m dimension vectors $v_{i}^{\left(m\right)}=\left[u_{i},u_{i+1},\ldots ,u_{i+m-1}\right]$, 1≤i≤N−m+1. The m dependence of functions are

$$C_{i}^{m}\left(r\right)={\left(N-m+1\right)}^{-1}\sum_{j=1}^{N-m+1}\theta (r-∥v_{i}^{\left(m\right)}-v_{j}^{\left(m\right)}∥)$$

and

$$C^{m}\left(r\right)={\left(N-m+1\right)}^{-1}\sum_{i=1}^{N-m+1}C_{i}^{m}\left(r\right)$$

where $∥v_{i}-v_{j}∥$ is Euclidean metric $∥v_{i}-v_{j}∥={\left(\sum_{h=0}^{m-1}{\left(u_{i+h}-u_{j+h}\right)}^{2}\right)}^{\frac{1}{2}}$ and θ(·) is Heaviside step function. $K_{2}$ entropy is defined as

$$K_{2}\;\mathrm{entropy}=\lim_{r\to 0}\lim_{m\to \infty }\lim_{N\to \infty }\frac{1}{\delta }\log\frac{C^{m}\left(r\right)}{C^{m+1}\left(r\right)}.$$

By incorporating the embedding vector based phase space reconstruction idea proposed by Takens [16] and replacing the Euclidean metric with the Chebyshev metric $∥v_{i}-v_{j}∥=\max_{h=0}^{m-1}\left|u_{i+h}-u_{j+h}\right|$, Eckmann and Ruelle [17] proposed an estimate of the K-S entropy through the so-called E-R entropy:

$$\Phi^{m}\left(r\right)={\left(N-m+1\right)}^{-1}\sum_{i=1}^{N-m+1}\log C_{i}^{m}\left(r\right)$$

$$\mathrm{E-R}\;\mathrm{entropy}=\lim_{r\to 0}\lim_{m\to \infty }\lim_{N\to \infty }\frac{1}{\delta }\left[\Phi^{m}\left(r\right)-\Phi^{m+1}\left(r\right)\right],$$

where the delay is often set to be δ=1.

The E-R entropy has been useful in classifying low-dimensional chaotic systems, but it becomes infinity for a process with superimposed noise of any magnitude [18]. Pincus [4] then extended the E-R entropy into the now well-known ApEn depending on a given embedding window length *m* and a distance cutoff *r* for the Heaviside function:

$$ApEn\left(U;m,r\right)=\Phi^{m}\left(r\right)-\Phi^{m+1}\left(r\right),$$

and

$$ApEn\left(m,r\right)=\lim_{N\to \infty }ApEn\left(U;m,r\right),\;N\;\mathrm{is}\;\mathrm{the}\;\mathrm{length}\;\mathrm{of}\;\mathrm{discrete}\;\mathrm{signal}\;U.$$

SampEn was proposed by Richman and Moorman [19] as an extension of ApEn to avoid the bias induced by countering the self-matching of each of the embedding vectors. Specifically, SampEn is formulated by:

$$B_{i}^{m}\left(r\right)={\left(N-m-1\right)}^{-1}\sum_{j=1,j\neq i}^{N-m}\theta (r-∥v_{i}^{\left(m\right)}-v_{j}^{\left(m\right)}∥),$$

$$B^{m}\left(r\right)={\left(N-m\right)}^{-1}\sum_{i=1}^{N-m}B_{i}^{m}\left(r\right),$$

$$A_{i}^{m}\left(r\right)={\left(N-m-1\right)}^{-1}\sum_{j=1,j\neq i}^{N-m}\theta (r-∥v_{i}^{\left(m+1\right)}-v_{j}^{\left(m+1\right)}∥),$$

$$A^{m}\left(r\right)={\left(N-m\right)}^{-1}\sum_{i=1}^{N-m}A_{i}^{m}\left(r\right),$$

$$SampEn\left(U;m,r\right)=-\log\frac{A^{m}\left(r\right)}{B^{m}\left(r\right)},\;\mathrm{fix}\;m\;\mathrm{and}\;r,$$

$$SampEn\left(m,r\right)=\lim_{N\to \infty }SampEn\left(U;m,r\right),\;N\;\mathrm{is}\;\mathrm{the}\;\mathrm{length}\;\mathrm{of}\;\mathrm{discrete}\;\mathrm{signal}\;U.$$

The coarse-graining multi-scale entropy-based complexity measurement can be traced back to the work by Zhang [20] and Fogedby [21]. In [1,22] Costa et al. calculated entropy at each coarse-grained scale using SampEn and named this process as the MSE. As commented by Nikulin and Brismar [10], a problem of the MSE algorithm is the use of the same matching criterion *r* for all scales, which causes systematic bias to SampEn.

## 3. The Systematic Bias of Entropy Calculation in MSE

In MSE [1,22], the embedding vector matching threshold *r* in defined by the standard deviation of the original signal. Using the same threshold, entropy of Gaussian signal decreases with the scale used to downsample the original signal. By contrast, entropy of 1/f signal remains unchanged when scale increases. As 1/f signal is known to have high complexity while Gaussian noise has a very low complexity, the monotonic MSE decaying trend or the sum of MSE at different scales were proposed as a metric for quantifying signal complexity.

However, the moving-average based coarse-graining process automatically scales down the subsampled signal at different time scales. Without correction, this additional multiplicative scaling will be propagated into the standard deviation of the signal to be assessed at each time scale and will artificially change sample entropy. This bias can be easily seen from the coarse-graining of a Gaussian noise.

Denote a Gaussian variable and its observations by $X=\{x_{1},x_{2},\ldots ,x_{N}\}$, where *N* indicates the length of the time series. The coarse-graining or moving averaging process can be described by $Y^{\left(\tau \right)}=\left\{y_{j}^{\left(\tau \right)}\right\}$, $y_{j}^{\left(\tau \right)}=1/\tau \sum_{i=(j-1)\tau +1}^{j\tau }x_{i}$ where τ>0 is the coarse-graining level or the so-called “scale”. Given the mutual independence of the individual samples of *X*, the moving averaging of these samples can be considered as an average of independent random variables rather than observations of a particular random variable. In other word, we can rewrite $Y^{\left(\tau \right)}$ to be $Y_{j}^{\left(\tau \right)}=1/\tau \sum_{i=(j-1)\tau +1}^{j\tau }X_{i}$, where $X_{i}$ is a random variable. For Gaussian noise *X*, $X_{i}$ will be Gaussian noise too and can be fully characterized with the same mean μ and standard deviation (SD) σ. Through a simple mathematics operation, we can get that $\mathrm{SD}\left(Y^{\left(\tau \right)}\right)=\sigma /\sqrt{\tau }$. Because SD(τ) monotonically decreases with τ, if we do not adjust the matching threshold, the number of matched embedded vectors will increase with τ, resulting a decreasing SampEn.

Entropy of a Gaussian distributed variable can be calculated through Shannon entropy:

$$\begin{array}{ccc} & & H\left(Y\right)=-\int_{-\infty }^{+\infty }p(y)\log p(y)\;dy\\ & & =-\int_{-\infty }^{+\infty }p\left(y\right)\log\left(\frac{1}{\sigma_{y}\sqrt{2\pi }}e^{-\frac{{\left(y-\mu_{y}\right)}^{2}}{2\sigma_{y}^{2}}}\right)\;dy\\ & & =-\int_{-\infty }^{+\infty }p\left(y\right)\log\left(\frac{1}{\sigma_{y}\sqrt{2\pi }}\right)\;dy-\int_{-\infty }^{+\infty }p\left(y\right)\log\left(e^{-\frac{{\left(y-\mu_{y}\right)}^{2}}{2\sigma_{y}^{2}}}\right)\;dy\\ & & =-\log\left(\frac{1}{\sigma_{y}\sqrt{2\pi }}\right)\int_{-\infty }^{+\infty }p(y)\;dy+\frac{1}{2\sigma_{y}^{2}}\int_{-\infty }^{+\infty }{\left(y-\mu_{y}\right)}^{2}p\left(y\right)\;dy\\ & & =\frac{1}{2}\log\left(2\pi \sigma_{y}^{2}\right)+\frac{1}{2}. \end{array}$$

For the simplicity of description, we often normalize the random variable to have a μ=0 and σ=1. Considering the scale-dependent SD derived above, we can then get the Shannon entropy of the Gaussian variable at the scale τ by

$$H\left(Y^{\left(\tau \right)}\right)=\frac{1}{2}\log\left(\frac{2\pi }{\tau }\right)+\frac{1}{2}$$

This equation clearly demonstrates the non-linearly but monotonically decreasing relationship of entropy with respect to scale τ.

Below, we provided mathematical derivation of the dependence of MSE on the signal subsampling scale. Given the *m* dimensional embedding vectors $Z_{j}^{\left(m\right)}=\left[Y_{j},Y_{j+1},\ldots ,Y_{j+m-1}\right]$, sample entropy can be expressed as [22]

$$\begin{array}{ccc} & & SampEn\left(Y;m,r\right)=-\log\frac{\mathrm{Pr}(∥Z_{j}^{\left(m+1\right)}-Z_{i}^{\left(m+1\right)}∥\leq r)}{\mathrm{Pr}(∥Z_{j}^{\left(m\right)}-Z_{i}^{\left(m\right)}∥\leq r)}\\ & & =-\log\mathrm{Pr}\left(∥Z_{j}^{\left(m+1\right)}-Z_{i}^{\left(m+1\right)}∥\leq r|∥Z_{j}^{\left(m\right)}-Z_{i}^{\left(m\right)}∥\leq r\right). \end{array}$$

where ∥·∥ is the Chebyshev distance.

For m=1, we can have

$$\{∥Z_{j}^{\left(m\right)}-Z_{i}^{\left(m\right)}∥\leq r\}=\{|Y_{j}-Y_{i}\left|\leq r\right\},$$

and

$$\begin{array}{ccc} & & \{∥Z_{j}^{\left(m+1\right)}-Z_{i}^{\left(m+1\right)}∥\leq r\}=\{\max\{|Y_{j}-Y_{i}\left|,\right|Y_{j+1}-Y_{i+1}\left|\}\leq r\right\}\\ & & =\left\{\right|Y_{j}-Y_{i}\left|\leq r\}\wedge \{\right|Y_{j+1}-Y_{i+1}\left|\leq r\right\}. \end{array}$$

Thus,

$$\begin{array}{ccc} & & SampEn\left(Y;m,r\right)=-\log\frac{\mathrm{Pr}(\{|Y_{j}-Y_{i}\left|\leq r\}\wedge \{\right|Y_{j+1}-Y_{i+1}\left|\leq r\}\right)}{\mathrm{Pr}(|Y_{j}-Y_{i}|\leq r)}\\ & & =\mathrm{Pr}\left(|Y_{j+1}-Y_{i+1}|\leq r||Y_{j}-Y_{i}|\leq r\right). \end{array}$$

Based on the iid condition of $Y_{j}$, we can draw a conclusion that

$$\mathrm{Pr}\left(|Y_{j+1}-Y_{i+1}|\leq r||Y_{j}-Y_{i}|\leq r\right)=\mathrm{Pr}\left(|Y_{j+1}-Y_{i+1}|\leq r\right).$$

If m≥2, we can get

$$\{∥Z_{j}^{\left(m\right)}-Z_{i}^{\left(m\right)}∥\leq r\}=\{\max_{k\in \{0,\ldots ,m-1\}}\left\{\right|Y_{j+k}-Y_{i+k}|\}\leq r\},$$

and

$$\begin{array}{ccc} & & \{∥Z_{j}^{\left(m+1\right)}-Z_{i}^{\left(m+1\right)}∥\leq r\}=\{\max_{k\in \{0,...,m\}}\left\{\right|Y_{j+k}-Y_{i+k}\left|\}\leq r\right\}\\ & & =\left\{\right|Y_{j+m}-Y_{i+m}|\leq r\}\wedge \{\max_{k\in \{0,...,m-1\}}\left\{\right|Y_{j+k}-Y_{i+k}|\}\leq r\}. \end{array}$$

Therefore,

$$\begin{array}{ccc} & & SampEn(Y;m,r)\\ & & =-\log\frac{\left\{\right|Y_{j+m}-Y_{i+m}|\leq r\}\wedge \{\max_{k\in \{0,...,m-1\}}\left\{\right|Y_{j+k}-Y_{i+k}\left|\}\leq r\right\}}{\{\max_{k\in \{0,...,m-1\}}\left\{\right|Y_{j+k}-Y_{i+k}\left|\}\leq r\right\}}\\ & & =\mathrm{Pr}\left(|Y_{j+m}-Y_{i+m}|\leq r|\{\max_{k\in \{0,...,m-1\}}\left\{\right|Y_{j+k}-Y_{i+k}\left|\}\leq r\right\}\right). \end{array}$$

and

$$\mathrm{Pr}\left(|Y_{j+m}-Y_{i+m}|\leq r|\{\max_{k\in \{0,\ldots ,m-1\}}\left\{\right|Y_{j+k}-Y_{i+k}\left|\}\leq r\right\}\right)=\mathrm{Pr}\left(|Y_{j+m}-Y_{i+m}|\leq r\right),$$

given the mutual independence of $Y_{j}$. It should be noted that this conclusion does not require the condition of identical distribution, as long as the condition of independence is sufficient.

For the simplicity of description, we re-denote $Y_{j+m}$ and $Y_{i+m}$ by two general normally distributed but independent random variables ξ and η whose means are 0 and SDs are 1. The joint probability density functions (PDF) is

$$p\left(\xi ,\eta \right)=\frac{1}{2\pi \sigma_{y}^{2}}e^{-\frac{{\left(\xi -\mu_{y}\right)}^{2}+{\left(\eta -\mu_{y}\right)}^{2}}{2\sigma_{y}^{2}}}$$

and probability is

$$\begin{array}{ccc} & & \mathrm{Pr}\left(|\xi -\eta |\leq r\right)=\underset{|\xi -\eta |\leq r}{\;\int \int \;}\frac{1}{2\pi \sigma_{y}^{2}}e^{-\frac{{\left(\xi -\mu_{y}\right)}^{2}+{\left(\eta -\mu_{y}\right)}^{2}}{2\sigma_{y}^{2}}}\;d\xi \;d\eta \\ & & =\int_{-\infty }^{\infty }\int_{\eta -r}^{\eta +r}\frac{1}{2\pi \sigma_{y}^{2}}e^{-\frac{{\left(\xi -\mu_{y}\right)}^{2}+{\left(\eta -\mu_{y}\right)}^{2}}{2\sigma_{y}^{2}}}\;d\xi \;d\eta . \end{array}$$

We can then get

SampEn(Y;m,r)=−logPr|Yj+1−Yi+1|≤r=−logPr|ξ−η|≤r=−log12πσy2∫−∞∞∫η−rη+re−(ξ−μy)2+(η−μy)22σy2dξdηt=η−μyσys=ξ−μyσy−log12π∫−∞∞∫t−rσyt+rσye−s2+t22dsdt.

Similar to Shannon entropy calculating, after normalize the random variable to have a μ=0 and σ=1, the scale-dependent SD derived for coarse grained signal is $SD\left(Y^{\left(\tau \right)}\right)=1/\sqrt{\tau }$. We can get

$$SampEn\left(Y^{\left(\tau \right)};m,r\right)=-\log\left(\frac{1}{2\pi }\int_{-\infty }^{\infty }\int_{t-r\sqrt{\tau }}^{t+r\sqrt{\tau }}e^{-\frac{s^{2}+t^{2}}{2}}\;ds\;dt\right).$$

Since the interval $\left[t-r\sqrt{\tau },t+r\sqrt{\tau }\right]$ increases with τ, the above integral monotonically increases with τ. Accordingly, the negative logarithm based sample entropy $SampEn(Y^{\left(\tau \right)};m,r)$ will monotonically decreases with τ. This is consistent with the aforementioned Shannon entropy-based MSE bias description.

The systematic bias in MSE can be corrected by using a scale adaptive matching threshold. One approach to adjust the threshold is to use $SD^{\left(\tau \right)}=SD\left(0\right)/\sqrt{\tau }$ for scale τ during $SampEn(Y^{\left(\tau \right)};m,r)$ calculation. This works well for Gaussian signal but may not be effective for other signals if they have extra scale-dependent SD behavior in addition to that induced by the subsampling scale. Finding the theoretical scale-dependent SD equation may not be trivial too. Instead, SD can be directly calculated from the data after each coarse graining. This approach has been proposed in [10].

To demonstrate the systematic bias of MSE and the effeteness of the correction method, we used three synthetic time series with known entropy difference: the Gaussian noise, a 1/f noise, and a random walk. The length of time series was $N=2\times 10^{4}$. MSE with and without bias correction were performed.

## 4. Results

**Figure 1:**
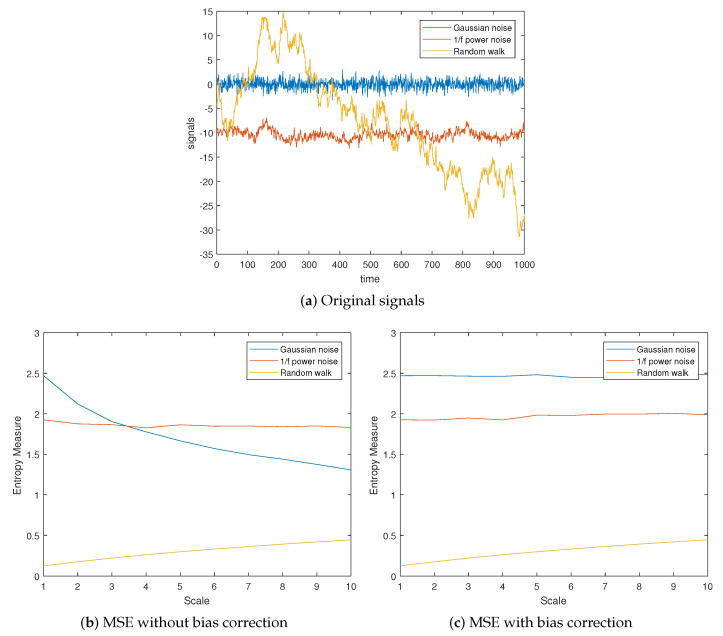
Different signals and their SampEn calculated at different scales. (**a**) The original signals before coarse graining; (**b**) MSE without bias correction; (**c**) MSE with bias correction.

Figure 1 shows the results of MSE with and without bias correction for the three time series (Figure 1a). Parameters used for SampEn calculation were m=2, and r=0.15×SD. Without bias correction, MSE produced a monotonically decaying SampEn for Gaussian noise when scale increases. By contrast, SampEn of Gaussian noise stays the same level at different scales after bias correction. The SD bias showed minor effects on SampEn calculation for both 1/f noise and the random walk. Correcting the bias did not dramatically change the SampEn at different scales.

## 5. Discussion and Conclusions

We provided a full mathematical derivation for the systematic bias in MSE introduced by the coarse graining process. We then used synthetic data to show the bias and the correction of it using dynamic SD calculation. Bias correction for Gaussian data MSE calculation works exactly as described by the theoretical descriptions given in this paper. The systematic bias does not appear to be a big issue for the temporally correlated process such as the 1/f noise and random walk. This is because variance of a temporally correlated process does change with the subsampling process if the sampling rate is still higher than the maximal frequency. According to [23], both 1/f noise and random work can be considered special cases of the autoregressive integrated moving average (ARIMA) model. As we derived in Appendix A, an ARIMA model is still an ARIMA model after coarse graining given the condition of that the residuals at different time points are independently and identically distributed (i.i.d.) Gaussian noise. In other words, the moving averaging process will not change the signal variance and will not change SampEn.

While we only showed the results based on one particular set SampEn calcualtion parameters, we included additional figures in Appendix B showing that the bias and the bias correction are still true for other parameters. We did not show the effects of bias correction on real data, but the results shown in the synthetic data should be generalizable to real applications since both the math derivations and the correction process are independent of any specific data but rather general to any dynamic system.

## Figures and Tables

**Figure A1:**
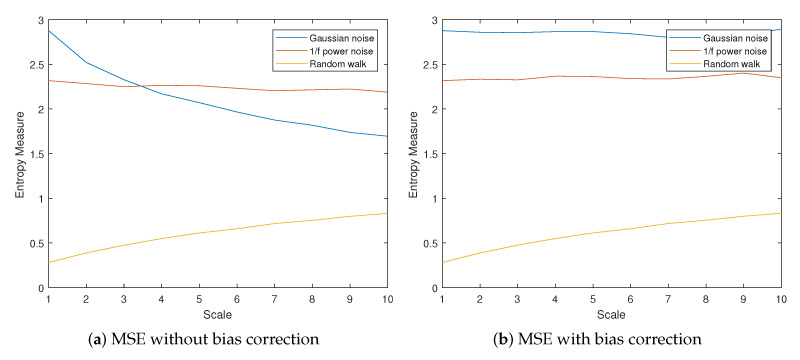
MSE calculation results with m=2 and r=0.1×SD. Data length $N=2\times 10^{4}$.

**Figure A2:**
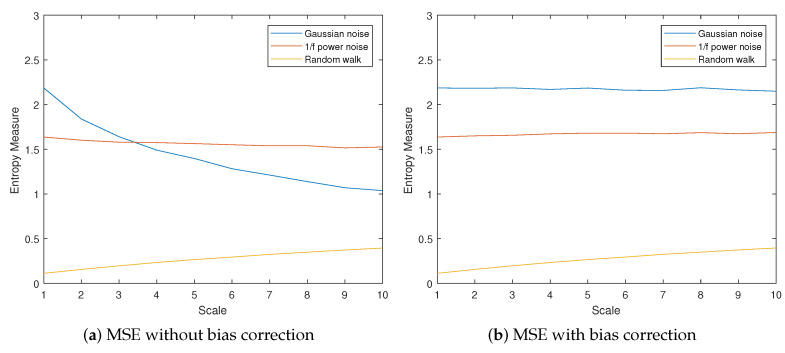
MSE calculation results with m=2 and r=0.2×SD. Data length $N=2\times 10^{4}$.

**Figure A3:**
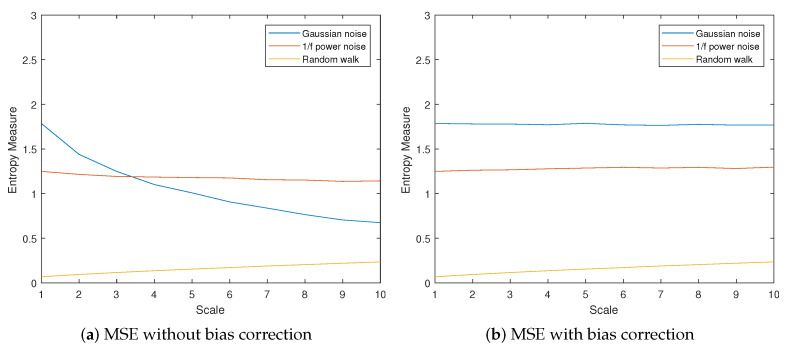
MSE calculation results with m=2 and r=0.3×SD. Data length $N=2\times 10^{4}$.

**Figure A4:**
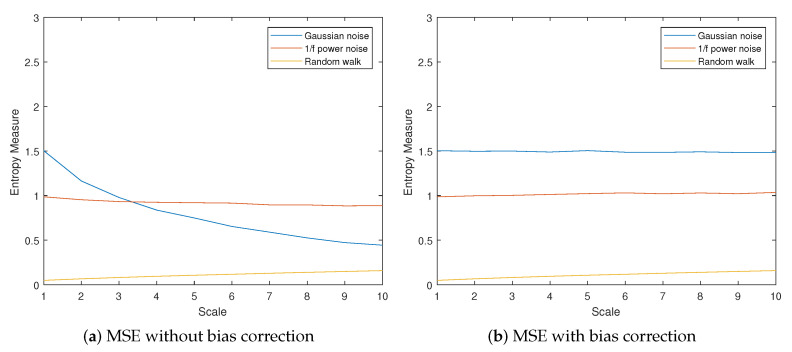
MSE calculation results with m=2 and r=0.4×SD. Data length $N=2\times 10^{4}$.

**Figure A5:**
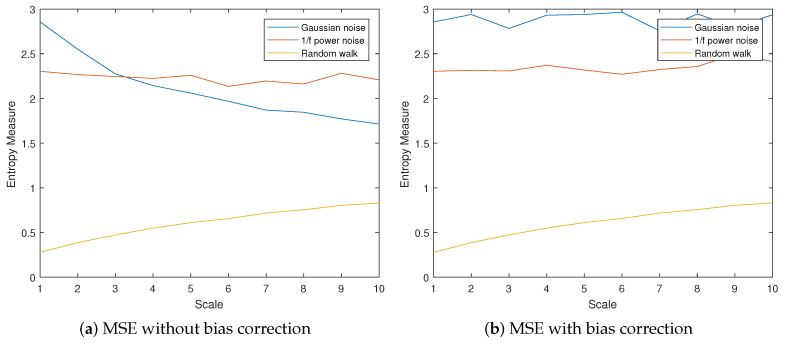
MSE calculation results with m=3 and r=0.1×SD. Data length $N=2\times 10^{4}$.

**Figure A6:**
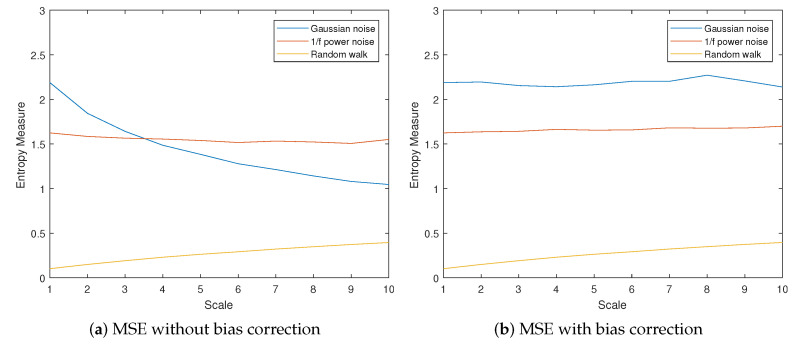
MSE calculation results with m=3 and r=0.2×SD. Data length $N=2\times 10^{4}$.

**Figure A7:**
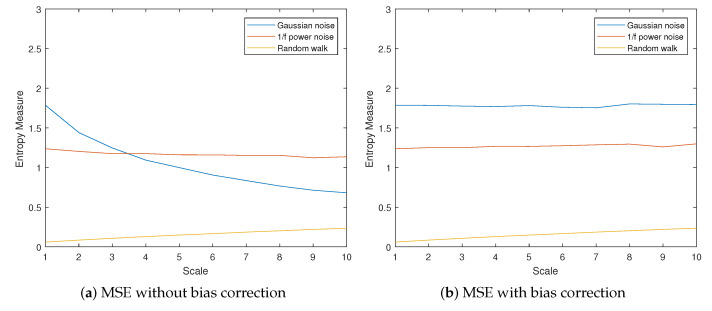
MSE calculation results with m=3 and r=0.3×SD. Data length $N=2\times 10^{4}$.

**Figure A8:**
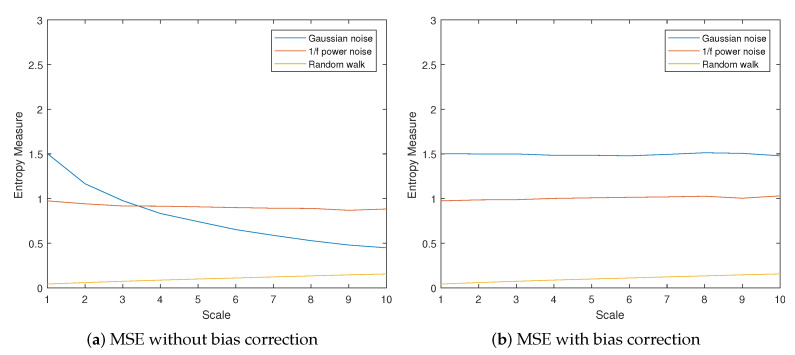
MSE calculation results with m=3 and r=0.4×SD. Data length $N=2\times 10^{4}$.

## Abbreviations

The following abbreviations are used in this manuscript:

MSE	multi-scale sample entropy
SampEn	sample entropy
ApEn	Approximate entropy
ARIMA	autoregressive integrated moving average

## Appendix A The Coarse-Grained ARIMA Process

Assume that a time series $\left\{X_{i}\right\}$ can be modeled by an ARIMA process: ARIMA(p,d,q):

$$\left(1-\sum_{h=1}^{p}\varphi_{h}L^{h}\right){\left(1-L\right)}^{d}X_{i}=\left(1+\sum_{h=1}^{q}\theta_{h}L^{h}\right)\varepsilon_{i},$$

where $\left\{\varepsilon_{i}\right\}$ are i.i.d. Gaussian noise, *L* is the lag operator.

Denote the consecutive coarse-grained time series of $\left\{X_{i}\right\}$ by $\left\{Y_{j}^{\left(\tau \right)}\right\}$:

$$Y_{j}^{\left(\tau \right)}=\frac{1}{\tau }\sum_{i=(j-1)\tau +1}^{j\tau }X_{i}.$$

where τ is scale.

Let

$$\epsilon_{j}^{\left(\tau \right)}=\frac{1}{\tau }\sum_{i=(j-1)\tau +1}^{j\tau }\varepsilon_{i},$$

then $\left\{\epsilon_{j}^{\left(\tau \right)}\right\}$ are also i.i.d. Gaussian and we can have

$$\begin{array}{ccc} & & \left(1-\sum_{h=1}^{p}\varphi_{h}L^{h}\right){\left(1-L\right)}^{d}\left(\frac{1}{\tau }\sum_{i=(j-1)\tau +1}^{j\tau }X_{i}\right)\\ & & =\frac{1}{\tau }\sum_{i=(j-1)\tau +1}^{j\tau }\left[\left(1-\sum_{h=1}^{p}\varphi_{h}L^{h}\right){\left(1-L\right)}^{d}X_{i}\right]\\ & & =\frac{1}{\tau }\sum_{i=(j-1)\tau +1}^{j\tau }\left[\left(1+\sum_{h=1}^{q}\theta_{h}L^{h}\right)\varepsilon_{i}\right]\\ & & =\left(1+\sum_{h=1}^{q}\theta_{h}L^{h}\right)\left(\frac{1}{\tau }\sum_{i=(j-1)\tau +1}^{j\tau }\varepsilon_{i}\right). \end{array}$$

and

$$\left(1-\sum_{h=1}^{p}\varphi_{h}L^{h}\right){\left(1-L\right)}^{d}Y_{j}^{\left(\tau \right)}=\left(1+\sum_{h=1}^{q}\theta_{h}L^{h}\right)\epsilon_{j}^{\left(\tau \right)}.$$

This proves that for any scale τ, $\left\{Y_{j}^{\left(\tau \right)}\right\}$ is also an ARIMA(p,d,q) process.

## Appendix B Numerical Results on Different *m* and *r*

The following Figure A1, Figure A2, Figure A3, Figure A4, Figure A5, Figure A6, Figure A7 and Figure A8 show additional MSE calculation results for different SampEn parameters m and r with and without bias correction. N means the length of the time series at scale 1. Theses figures confirmed the systematic bias in the original MSE algorithm for different m and r and the data adaptive correction successfully removed the bias for all assessed signals.

## References

[1] Costa M., Goldberger A.L., Peng C.K. (2002). Multiscale entropy analysis of complex physiologic time series. Phys. Rev. Lett..

[2] Lake D.E., Richman J.S., Griffin M.P., Moorman J.R. (2002). Sample entropy analysis of neonatal heart rate variability. Am. J. Physiol. Regul. Integr. Comp. Physiol..

[3] Delgado-Bonal A., Marshak A. (2019). Approximate entropy and sample entropy: A comprehensive tutorial. Entropy.

[4] Pincus S.M. (1991). Approximate entropy as a measure of system complexity. Proc. Natl. Acad. Sci. USA.

[5] Alcaraz R., Rieta J.J. (2010). A review on sample entropy applications for the non-invasive analysis of atrial fibrillation electrocardiograms. Biomed. Signal Process. Control.

[6] Wang Z., Li Y., Childress A.R., Detre J.A. (2014). Brain entropy mapping using fMRI. PLoS ONE.

[7] Sokunbi M.O. (2014). Sample entropy reveals high discriminative power between young and elderly adults in short fMRI data sets. Front. Neuroinform..

[8] Richman J.S., Lake D.E., Moorman J.R. (2004). Sample entropy. Methods Enzymol..

[9] Humeau-Heurtier A. (2015). The multiscale entropy algorithm and its variants: A review. Entropy.

[10] Nikulin V.V., Brismar T. (2004). Comment on “Multiscale entropy analysis of complex physiologic time series”. Phys. Rev. Lett..

[11] Nyquist H. (1924). Certain factors affecting telegraph speed. Trans. Am. Inst. Electr. Eng..

[12] Hartley R.V. (1928). Transmission of information 1. Bell Syst. Tech. J..

[13] Shannon C.E. (1948). A mathematical theory of communication. Bell Syst. Tech. J..

[14] Sinai Y.G. (1959). On the notion of entropy of a dynamical system. Dokl. Russ. Acad. Sci..

[15] Grassberger P., Procaccia I. (1983). Estimation of the Kolmogorov entropy from a chaotic signal. Phys. Rev. A.

[16] Takens F. (1983). Invariants related to dimension and entropy. Atas Do.

[17] Eckmann J., Ruelle D. (1985). Ergodic theory of chaos and strange attractors. Rev. Mod. Phys..

[18] Pincus S.M., Gladstone I.M., Ehrenkranz R.A. (1991). A regularity statistic for medical data analysis. J. Clin. Monit..

[19] Richman J.S., Moorman J.R. (2000). Physiological time-series analysis using approximate entropy and sample entropy. Am. J. Physiol. Heart Circ. Physiol..

[20] Zhang Y.C. (1991). Complexity and 1/f noise. A phase space approach. J. De Phys. I.

[21] Fogedby H.C. (1992). On the phase space approach to complexity. J. Stat. Phys..

[22] Costa M., Goldberger A.L., Peng C.K. (2005). Multiscale entropy analysis of biological signals. Phys. Rev. E.

[23] Stadnitski T. (2012). Measuring fractality. Front. Physiol..

